# “*Living their best life*”: PhotoVoice insights on well-being, inclusion, and access to public spaces among adolescent refugee girls in urban resettlement

**DOI:** 10.1080/17482631.2024.2431183

**Published:** 2024-12-09

**Authors:** Alli Gillespie, Zahyyeh Abu-Rubieh, Lily Coll, Manar Matti, Carine Allaf, Ilana Seff, Lindsay Stark

**Affiliations:** a Brown School at Washington University in St. Louis, St. Louis, MO, USA; b Independent Consultant, Chicago, IL, USA; c Qatar Foundation International, Washington, DC, USA

**Keywords:** PhotoVoice, MENA, refugees, adolescents, participatory methods, intersectionality, well-being, belonging, public spaces, Arabic

## Abstract

**Purpose:**

Newcomer adolescent girls from the Middle East and North Africa region face intersectional challenges and opportunities upon resettlement. This study employs PhotoVoice participatory research methodology to explore perspectives on well-being and belonging shared by six students who resettled to Chicago from Iraq and Syria.

**Methods:**

Two programme sessions consisted of participants reflecting on their photographic responses to four prompts in focus group discussions. The subsequent four sessions included qualitative analysis skill building, participant-led generation of thematic codes and diagrams using their data, and the creation of action plan posters to share back with the community. The research team then analysed all data using a grounded theory approach with constant comparative analysis.

**Results:**

Four major themes emerged: 1) public spaces served as a vehicle for exploring well-being and belonging; 2) intersectional inclusion in public spaces was deemed a vital priority; 3) schools held an important role in facilitating belonging and access to public spaces; and 4) language was a critical barrier and facilitator to access and inclusion.

**Conclusions:**

Findings highlight the need for holistic approaches to support refugee youth in urban contexts and emphasize the role of schools in facilitating inclusive access to public spaces to strengthen newcomer students’ well-being and belonging.

## Introduction

As of 2023, approximately 43.3 million children globally had experienced displacement due to conflicts and violence (UNICEF, 2023). As a result of this massive displacement, the U.S. resettlement programme resettled 31,800 refugees and asylum seekers within the first eight months of Fiscal Year 2023 (Batalova & Ward, 2023). Many of the refugees and asylum seekers resettled during the past decade arrived from countries in the Middle East and North Africa (MENA) region, with many forcibly displaced due to war post-9/11, economic instability, climate crises, and poor governance following the 2011 Arab Uprisings (Norman, 2023; Vine et al., 2020). In the years between 2013–2020, the U.S. resettled 100,754 refugees from Iraq and Syria, many of whom were children (UNHCR, 2024). These young individuals, representing a critical subset of the broader refugee population, have been exposed to a distinctive set of challenges from their flight to arrival in the United States, compounded by the intersection of adolescence and forced displacement (Lloyd, 2019). This intersection has presented unique complexities, as adolescents are, by nature, at a developmental stage marked by rapid cognitive, emotional, and social changes (Bronstein & Montgomery, 2011; Fazel et al., 2012). Upon their arrival in the United States, adolescent newcomers from the Middle East and North Africa (MENA) have continued to face the intricate challenge of simultaneously navigating the transformation from childhood to adulthood and adapting to a foreign culture.

The educational needs of newcomer students resettled to the U.S. are particularly pronounced, as immigrant and refugee youth must not only continue their learning in a new school system but also rapidly develop English language proficiency to communicate effectively with peers and access educational opportunities. Educational disparities, which may result from interrupted formal schooling in their countries of origin, further complicate this transition (Custodio & O’Loughlin, 2017). For example, MENA adolescents resettled in the U.S. often encounter school systems that treat heritage language as a problem to be solved by English-language programming, disregarding students’ rich linguistic skills–and cultural resources more broadly–and impacting their academic, social, and economic acculturation and well-being (Gillespie et al., 2022; Seff et al., 2024a). The cultural dislocation these students experience, exacerbated by differences in social norms, values, and cultural expectations, can induce a crisis of identity, whereby refugee adolescents grapple with reconciling their heritage with the demands of their new environment.

Moreover, many resettled adolescents from the MENA region must navigate mental health challenges shaped by experiences of violence, loss, and persecution in their countries of origin and potentially during other stages of migration and resettlement (Stark et al., 2022). The psychological ramifications of these experiences add another layer of complexity to adolescents’ adjustment and mental well-being (Mohamed & Thomas, 2017). A significant challenge arises from the psychological burden carried by many refugees, often manifesting in post-traumatic stress disorder (PTSD), depression, and anxiety (Lustig et al., 2004). These mental health issues are compounded by the process of acculturation, wherein adolescents must grapple with the pressures both to adapt to a new cultural context and to selectively shed, preserve, readopt, or reinterpret cultural resources from their countries of origin (Killian et al., 2018; Schwartz et al., 2010). This intricate interplay between mental health and acculturation becomes a pivotal concern in understanding the lives of resettled adolescent refugees. The impact of these challenges extends beyond individual well-being, as untreated mental health issues can hinder educational attainment, social integration, and overall adjustment, shaping the trajectory of their and their community’s lives in their host country (Mofatteh, 2020).

Despite adversities like acculturative stress, discrimination, and loss of identity, youth affected by forced displacement display remarkable resilience, understood as the capacity to withstand and recover from adversity (Bennouna et al., 2021; Peterson, 2018; Springer et al., 2023). Newcomer students have been shown to navigate challenges by leveraging individual and collective resources, including forming same-generation friendships that aid their integration and safeguard their well-being (Bennouna et al., 2021; Springer et al., 2023). Building social support networks within their communities, schools, and among peers who share similar experiences becomes a cornerstone of their coping strategy (Al-Shatanawi et al., 2023). Moreover, the role of the family, offering a sense of stability and continuity, is of central importance (From Crisis to Coping, 2023). Additionally, creative outlets such as art, music, sports, or other forms of self-expression serve as both cathartic mechanisms and sources of empowerment (Feen-Calligan, Grasser, Smigels, et al., 2023). These strengths and adaptive strategies are vital not only for mitigating mental health challenges but also for fostering a sense of self-efficacy and hope among adolescent refugees, facilitating their agency in the resettlement process (Tran et al., 2022).

The agency of newcomer youth in research efforts is equally important in facilitating well-being. A notable research gap in the study of adolescent refugees and newcomers in the U.S. lies in the dearth of participatory qualitative research that allows adolescents to derive their own collective meaning from their experiences in order to shape the discourse around acculturation and identity. This oversight is particularly pronounced among adolescent girls, who often confront unique challenges that are intricately linked to their gender, such as gender-based violence, cultural expectations, and barriers to education (Ekstrom, 1972; Gender, Culture and Expectations | UIC Today, *n.d.*; Gender-Based Violence (Violence Against Women and Girls), *2019*). Involving adolescent girls as active participants in the research process is essential for gaining a nuanced understanding of their lives, aspirations, and the specific challenges they face. Educators and academic institutions may lack detailed awareness and knowledge pertaining to the background and experiences of newcomer adolescent girls from the MENA region. Participatory qualitative research can offer a platform for these girls to voice their own narratives, enabling scholars, policymakers, and practitioners to address their needs more comprehensively and to implement more effective support structures. Further, evidence grounded in lived experience and participatory analysis can counter widespread, discriminatory narratives about newcomer populations, adding nuance to discourse and decision-making throughout the broader education system. Recognizing this research gap and advocating for participatory research is crucial for advancing our understanding of the experiences and perspectives of newcomer adolescent girls and, in turn, for shaping policies and interventions that empower and uplift this intersectionally-marginalized population.

This sub-study of the Study of Adolescent Lives after Migration to America (SALaMA) utilizes PhotoVoice participatory research methodology to explore the experiences and perspectives of newcomer adolescent girls from the MENA region resettled in Chicago, Illinois during the past 10 years. Further, it aims to illuminate the facilitators of well-being and belonging that they collectively prioritize as they confront the intersectional challenges and opportunities associated with adjusting to life in the United States. Adolescents represent the future of any society, and their experiences during resettlement have a profound impact on the evolving cultural, social, and economic landscapes of their communities. This research not only contributes to academic understandings of these dynamics but, more crucially, holds practical and policy-oriented significance. Practitioners and policymakers within the U.S. education system have a responsibility to prioritize efforts that centre refugee students’ intersectional lived experiences and stated support needs.

## Methods

### PhotoVoice and SALaMA EMPOWER

Findings from the current analysis are part of the broader Study of Adolescent Lives After Migration to America (SALaMA), a mixed-methods, multi-site study that aims to assess the psychosocial well-being of adolescent students resettled from the MENA region (Stark et al., 2020). Previous quantitative and qualitative findings suggest potential pathways to mitigate intersectional disparities for newcomer students and to promote their well-being (Bennouna et al., 2019; Gillespie et al., 2022; Seff et al., 2024b; Stark et al., 2022). Additionally, past analyses of focus groups and key informant interviews emphasize the pivotal role of schools in aiding adjustment and fostering a sense of belonging, advocating for culturally responsive pedagogies and equitable learning environments (Bennouna et al., 2019; Gillespie et al., 2022; Stark et al., 2021). Amidst these challenges, the importance of community and social integration emerges as a recurring theme among resettled refugee youth from the MENA region (Bennouna et al., 2021).

Data for the current paper were collected using PhotoVoice, a participatory, community-based methodology that uses photography to empower individuals to be active agents in qualitative research (Wang & Burris, 1997). As a methodology, PhotoVoice accounts for the idea that community members have a unique ability to capture spaces that may be unavailable to researchers (Wang & Burris, 1997). These recordings, in the form of photographs, are then utilized to promote critical dialogue about community strengths, concerns, and belonging (Wang & Burris, 1997). By training participants in photography, and subsequently qualitative data analysis, PhotoVoice prioritizes experiential learning alongside more traditional qualitative research methods of focus groups and discussion.

The SALaMA team utilized PhotoVoice in the present study due to its strength in application for the population of interest, resettled youth. For example, a previous PhotoVoice study conducted among refugee youth in the U.S. emphasized the protective role of same-generation friendships in mitigating negative health behaviours and shed light on how friendships can act as a buffer against challenges faced during resettlement (Springer et al., 2023). A second study that used PhotoVoice with Muslim adolescents in a Canadian high school highlighted the significance of this method for demonstrating to youth the importance of their perspectives and their capacity for enacting change (Miled, 2020). The social activism component of PhotoVoice, often a final public exhibition of the photographs, creates a platform for newcomer adolescent community members to uplift their voices and allows the study outcomes to be shared with a wider audience (Feen-Calligan, Grasser, Nasser, et al., 2023).

SALaMA’s EMPOWerment and rEsilience pRogram (EMPOWER) integrates traditional PhotoVoice techniques, including photography by participants, caption generation, and group reflection, complemented by qualitative analysis of these reflection sessions conducted with the participants (SALaMA, 2021). More details on how EMPOWER incorporated PhotoVoice methodology can be found in the SALaMA EMPOWER Facilitation Guide (SALaMA, 2021). While EMPOWER was first piloted in the Detroit Metropolitan Area (DMA), Michigan in 2021, the present study draws from its implementation in Chicago in the Spring of 2023.

### Program and participants

EMPOWER participants comprised six students in the Chicago Public School District (CPS). While CPS does not presently track their students’ migrant statuses, the school district keeps records of the number of newcomer students, defined as a student who is attending public school in the U.S. for the first time. The district ended the 2022–2023 school year with approximately 77,000 English learners, about one-quarter of all enrolled students (Nader et al., 2023). According to the CPS 2023–2024 demographics report, 24.7% of students are English Learners (CPS Demographics, 2023). CPS is an expansive and diverse system of 634 schools, with students from all over (CPS Demographics, 2023). However, the schools attended by study participants were ones that have historically and currently enrolled a larger number of resettled, newcomer students from the MENA region.

Three of the six participants were enrolled in one CPS high school (grades 9 through 12) and three participants were enrolled in two CPS elementary schools (grades pre-K through 8). Their ages ranged from 14 to 17, and all six participants identified as girls who used she/her pronouns. Recruitment was conducted using word-of-mouth, both through the personal and professional connections of one of the study facilitators and through partnership with a community-based refugee and immigrant support organization. Due to the word-of-mouth recruitment method, participants enrolled in the study in pairs–two pairs of close friends (Cece and Soso, and Jasmine and Julica), and one pair of cousins (Sarah and Victoria).^1^ Participants were all born in the MENA region and their length of stay in the U.S. ranged from one to nine years. Four of the participants immigrated from Syria, and two immigrated from Iraq.

Two co-facilitators led the study activities. One co-facilitator, a member of the SALaMA research team at Washington University in St. Louis, spoke English and was trained in social work and public health with refugee communities. The second co-facilitator, a SALaMA consultant and local community member, spoke Arabic and English, worked with families at a local refugee support organization, and had lived experience with the resettlement process in Chicago. Both co-facilitators contributed to the subsequent analysis and co-authored the manuscript.

Participation involved attending six sessions, which lasted about one and a half hours each. Sessions took place in a community room at a Chicago Public Library branch situated in close proximity to participants’ homes and schools on the north side of Chicago.

In the first session, co-facilitators shared four photo prompts with participants to guide their photography and reflection. While photo captions were optional, facilitators encouraged participants to accompany each photograph with a short description that exemplified its significance in response to the prompt. The prompts were: 1) What do you want people to know about you?, 2) What does well-being mean to you?, 3) How would you describe your life in the US to a friend back in your country of birth?, and 4) What does “belonging” mean to you? In the week between the first and second sessions, participants were asked to take photos responding to the prompts and to submit their photos and captions to the co-facilitators. The co-facilitators printed the photographs for participants to keep and added all photos and captions to a data slideshow to display and guide the following weeks of the programme.

In the subsequent two reflection sessions, facilitators opened discussion by presenting each participant’s chosen photo, organized by prompt. Conversations were not limited to content from the photo prompts, and discussion topics naturally progressed into themes such as identity, community, and family. The first reflection session included the full large group. The second reflection sessions were conducted in participant pairs, with two primarily in English and one primarily in Arabic, followed by a large group debrief to further reflect on and identify themes across the smaller groups.

The final three sessions involved training participants in qualitative coding analysis, using qualitative data visualization techniques, and, finally, creating products to communicate their results. In the first analysis session, co-facilitators guided participants through the process of coding their own transcripts from the previously completed focus groups. Participants practiced coding in small groups, using the photo prompts as a guiding framework. In the second analysis session, co-facilitators taught participants to utilize their new skills to diagram the codes, creating a visualization of the relationships between the fifteen codes participants felt were most critical for understanding the themes of their conversations. In the sixth and final session, participants worked in small groups to design action plan posters that communicated their shared priorities for change within their communities, based on their codes and diagrams. Following the EMPOWER programme sessions, participants’ photos, diagrams, and posters were displayed in a two-month exhibit at the community-based refugee and immigrant support organization and then presented in a gallery event at a CPS high school, which was attended by participants, family members, peers, and school and district staff.

### Ethical considerations

The study protocol was approved by the Washington University in St. Louis Institutional Review Board. Researchers obtained informed consent from participants’ parent or legal guardian prior to data collection, followed by verbal assent from participants themselves. The research team compensated participants with their choice of either $30 in cash or gift card, distributed during the last session of the programme along with a certificate of programme completion.

### Analysis

In addition to participants analysing their own transcript data, the research team conducted a separate analysis of the five transcripts from the two recorded reflection sessions. The research team developed a codebook, with codes gleaned inductively from the data, then cross-checked with the codes generated by participants themselves to ensure alignment (Table I). Three team members tested the codebook by applying codes to a subset of qualitative data, followed by a discussion of code application relevance and bias and subsequent codebook refinement. Using Dedoose analytical software, two team members coded the full dataset using the finalized codebook. Finally, the team reviewed coded excerpts using a grounded theory approach with constant comparative analysis (Chun Tie et al., 2019). Additionally, the team employed intersectionality as a critical lens of analysis, noting when participants’ experiences seemed to be influenced by the intersection of their identity dimensions and cross-cutting systems of marginalization in the U.S. context (Collins, 2019). The research team further triangulated the analysis of coded excerpts and photos with an analysis of participants’ code diagrams and action plan posters. Four prominent themes emerged from this multi-stage analysis and are explored in the following section.

**Table I.** Codebook with corresponding participant-generated codes.

Parent code	Child code	Corresponding participant-generated codes
Acculturation	*Navigating identity/perception of self*	
Affirming spaces		Safe places
Basic needs	*Cost of living*	Expensive
	*Food security*	
	*Housing security*	
	*Transportation access*	
Challenges/barriers		Anything difficult Lost
Community based support and resources		Care Community Helped Support
	*Arab-support network*	Arab communities
	*Service providers and authority figures*	Cops
Community dynamics	*Co-existence of diverse groups*	Neighbors
	*Community building/teamwork*	
	*Conflict resolution*	
	*Reception upon arrival*	
	*Social norms*	
Comparing life in US and country of origin		America
Demographic information/identity descriptors	*Age/grade*	Children Teenager Too young
	*Gender*	Women
	*Muslim*	Muslim
	*Nationality/country-of-origin*	
	*Race/skin color/ethnicity*	Skin colours
	*Refugee/migration status*	Refugee
	*Socioeconomic status*	
	*Special needs/disability*	Treatment of children with special needs
Environment, context, and infrastructure		Clean schools Place
	*Living beings*	
	*Outdoor public spaces*	Beach Going outside Park Water park
	*Season/weather/time of year*	
Feelings towards migration/resettlement	*Feelings towards people/community in the US*	
	*Perceptions of life in the US*	Attitude Point of view
	*Reminder of home/country of origin*	Back home
Gender roles and expectations		
Household dynamics	*Description of family members*	Family BrotherMomsParents
	*Family support and protection*	Protective Provide
	*Generational differences*	
	*Parent-child relationships*	
	*Rules, roles, and expectations*	Cleaning Cooking Control/يسيطر Order Responsibility Strict
	*Sibling relationships/birth order*	
Individual resources	*Academic motivation*	Learning
	*Achievements and accomplishments*	
	*Agency/autonomy*	
	*Alone time*	
	*Appreciation/acknowledgment of other cultures*	
	*Financial awareness*	Money
	*New experiences, growth, and potential*	Opportunities Wished
	*Resilience and coping*	
	*Self-confidence/self-esteem*	
	*Self-expression/presentation of identity*	
	*Sense of justice, accountability, fairness*	Attention Unequal Unfair Upstander
	*Social media*	
Interests and hobbies		Swimming
Language and literacy	*Arabic language loss*	
	*English language learning*	Speaking English
Mental health and psychosocial well-being	*Anger*	Angry
	*Anxiety/nerves/fear*	Scared Worried
	*Comfort/discomfort*	Annoying Uncomfortable
	*Feeling of freedom*	Freedom
	*Grief/loss/sadness*	Sad
	*Joy/happiness/fun/play/excitement*	Happy Having fun
	*Living one’s “best life”*	Best life
	*Other emotion*	Feelings Grateful Hate Lucky
	*Peace/calm/quiet*	
	*Sleep/rest*	
	*Support seeking*	Crazy
Movement/travel		
Other		Always Better Weak
Peer relationships	*Bullying/social isolation*	
	*Fighting/tension*	
	*Friendship*	
	*Peer support*	
	*Shared experiences*	
Relationship factors	*Belonging*	Belonging
	*Connection*	Connection
	*Dismissiveness/validation*	Does not care
	*Judgement*	Judgmental
	*Love*	
	*Maturity levels*	
	*Quality time*	
	*Respect*	Kindness Treatment
	*Trust*	
	*Understanding*	Listening Sympathy
Religion	*Beliefs and values*	
	*Celebration/holidays*	
	*Community*	
	*Institutions*	
	*Prayer and other practices*	
School based support and resources	*Family-school relationship*	
	*School initiatives and programming*	High school School
	*Student body composition*	
	*Teacher-student dynamics*	
Student name codes	*Cece*	
	*Jasmine*	
	*Julica*	
	*Sarah*	
	*Soso*	
	*Victoria*	
Violence	*Abuse and harrassment*	“Bomber” Mistreated
	*Discrimination/othering*	Different Discrimination Normal Offensive Sexist Weird
	*Safety and security*	Safe

## Results

Four major themes emerged from the data analysis: 1) public spaces as a vehicle for exploring well-being and belonging; 2) emphasis on intersectional inclusion in public spaces; 3) the role of schools in facilitating belonging and access to public spaces; and 4) language as a critical barrier and facilitator to access and inclusion. The following subsections examine each of these themes using participant perspectives, photos, and action plan posters.

### Public spaces as a vehicle for exploring well-being and belonging

**Figure 1.**
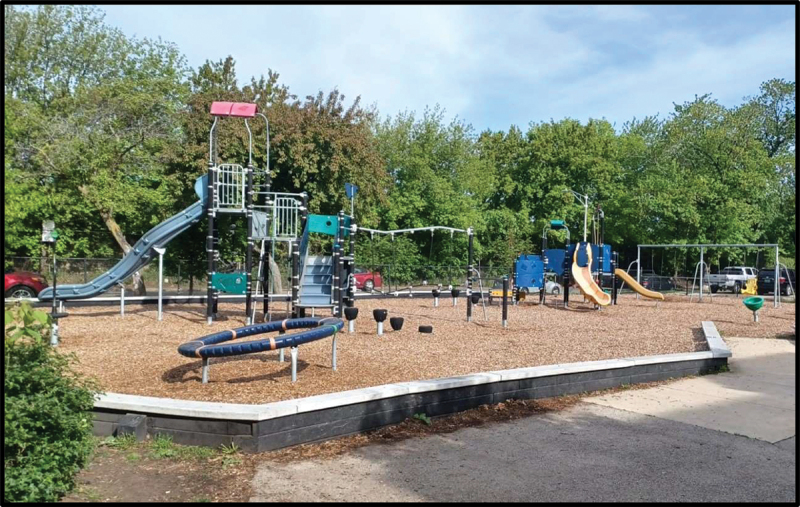
Julica, captioned “why I like it here so much,” submitted in response to the prompt “how would you describe your life in the US to a friend back in your country of birth?”.

**Figure 2.**
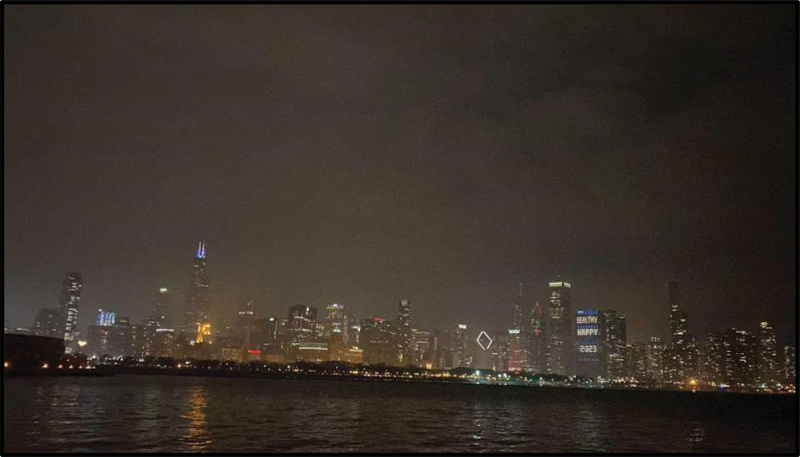
Victoria, submitted in response to the prompt “what do you want people to know about you?”.

**Figure 3.**
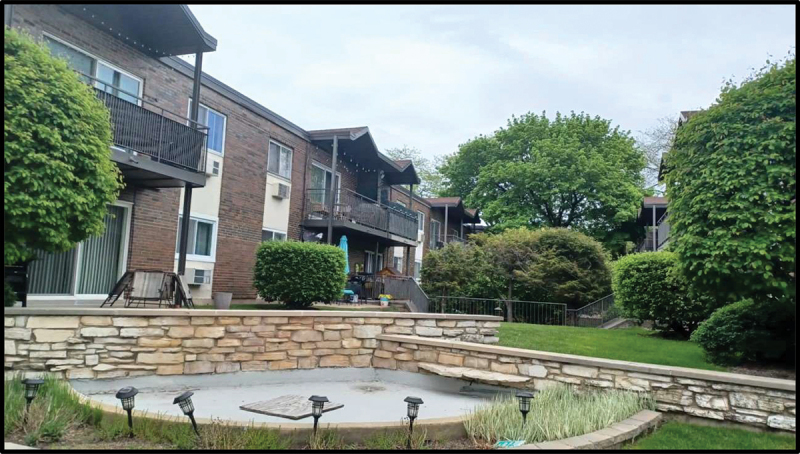
Julica, captioned “why I belong here,” submitted in response to the prompt “what does ‘belonging’ mean to you?”.

**Figure 4.**
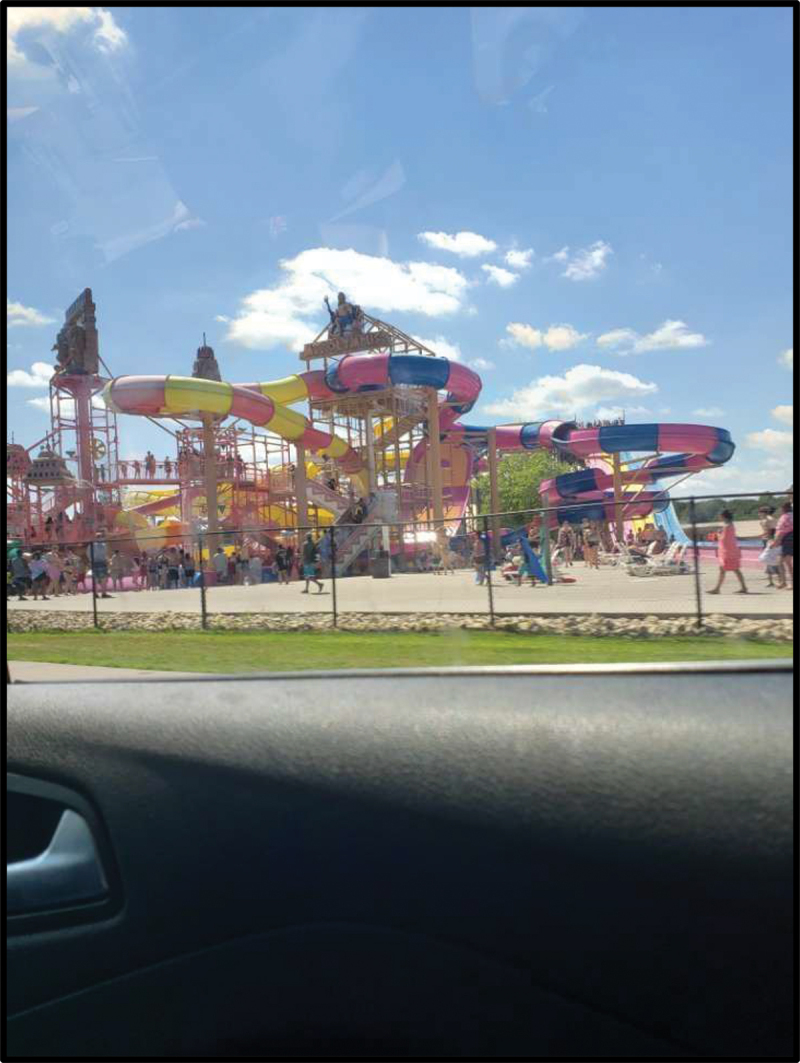
Sarah, captioned “nothing better than a water park on a sunny day,” submitted in response to the prompt “what does ‘belonging’ mean to you?”.

In participants’ photograph responses to the assigned prompts, as well as in the subsequent focus group discussions, the importance of nature and outdoor public spaces emerged as a prominent theme. In fact, all six participants’ photo responses to the prompt “How would you describe your life in the US to a friend back in your country of birth?” included some element of the outdoors. Examples included a photograph of an empty park surrounded by trees with the caption “Why I like it here so much” (Julia) (Figure 1) and a cityscape of the Chicago skyline seen from across the water (Victoria) (Figure 2). Similarly, photo responses to “What does belonging mean to you?” depicted themes of nature and the outdoors. Julica defined belonging with a photograph of her wooded backyard (Figure 3). Another participant, Sarah, brought a picture of an outdoor waterpark with the caption “Nothing better than a water park on a sunny day” (Figure 4). As PhotoVoice co-researchers, participants used the medium of photography and their descriptions of the public spaces they captured as a vehicle to explore their feelings and experiences of well-being and belonging.

**Figure 5.**
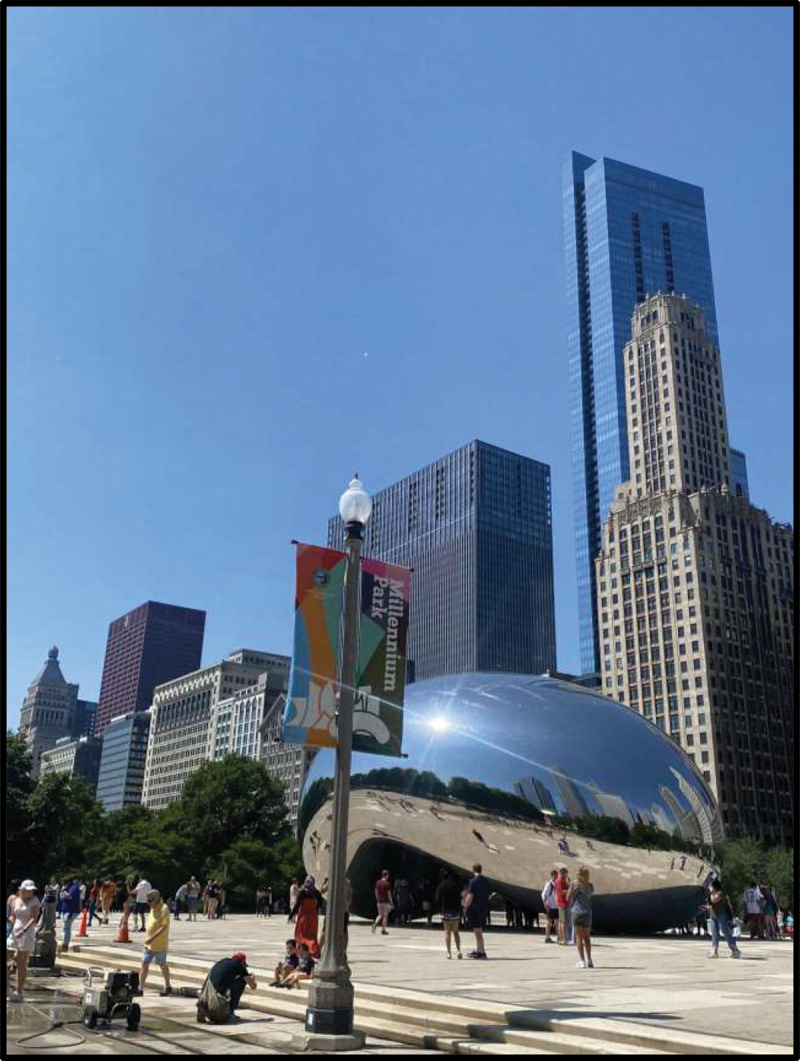
Victoria, submitted in response to the prompt “what does ‘belonging’ mean to you?”.

**Figure 6.**
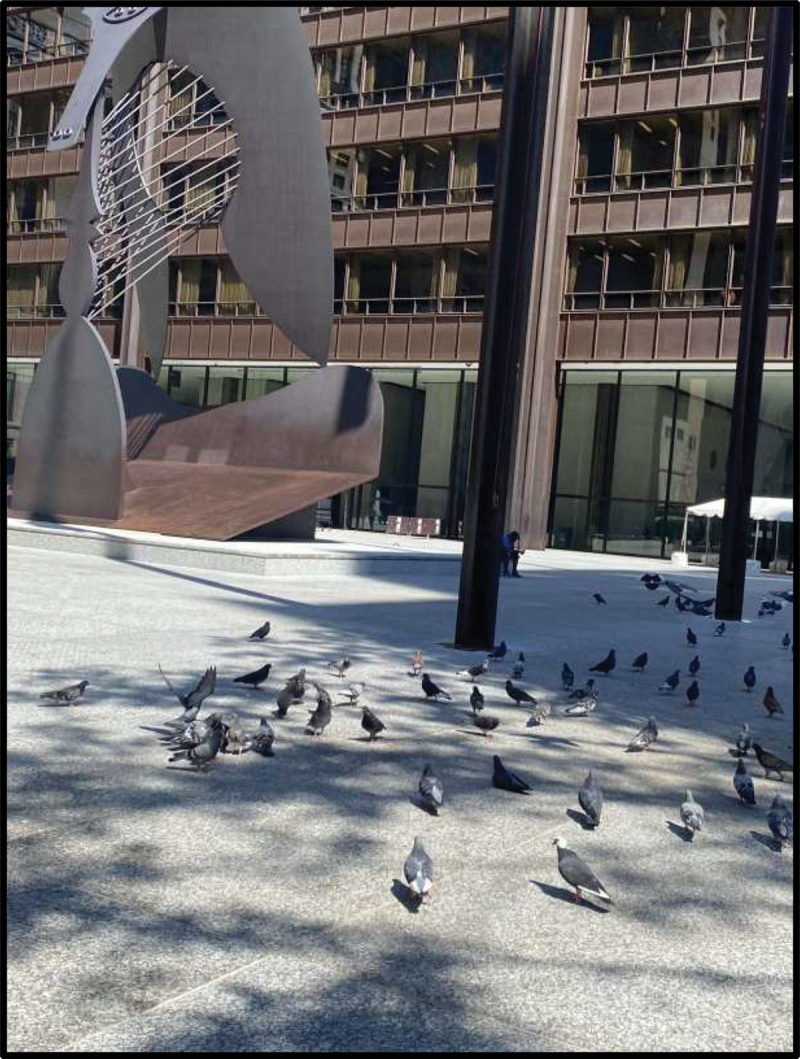
Victoria, submitted in response to the prompt “how would you describe your life in the US to a friend back in your country of birth?”.

Photographic motifs of nature and the outdoors were further elaborated upon throughout large and small group discussions, with participants emphasizing their association between outdoor public spaces and positive memories. In one discussion, Julica shared that the first thing that came to her mind when describing her life in the U.S. was a park, saying, “When I first came here, I started going to parks, then I started … going to beaches, barbecuing, and going to waterparks.” Victoria also portrayed outdoor public spaces when prompted to define belonging and to share how she would describe life in the U.S. with family back home; she showed a picture of the Chicago “Bean” sculpture (Figure 5) and said that she loved Millenium Park’s inclusion of “fun” activities such as a waterfall and an ice-skating rink. For the latter, she shared a picture of birds (Figure 6), noting that they represented belonging because they “remind me about these birds in my country, I used to have them and I used to feed them, and sometimes, I left them to fly, and they really loved it.”

Participants often contrasted their connection to public spaces in America with those from their home countries. While participants shared fond memories of life pre-migration, for example “[playing] in the streets because they were so empty” (Jasmine), they consistently described outdoor public spaces in the U.S. as more peaceful, clean, and quiet, building the affirming, comforting feeling they associated with being in these spaces. Julica explained, “There are also parks in Syria, but there are … parks that are extra good in America, and also you know, there are nice and clean beaches.” Another participant emphasized the peaceful aspect of public spaces in Chicago, potentially indicating the impact that resettling from a conflict-affected context had on her mental health and psychosocial well-being:

… I chose this picture cuz life here is like, so much more peaceful than how it was back in Iraq. As you see here, it’s like, so peaceful … Like in Iraq, there’s no parks, there’s just like swings but it’s all muddy and stuff so like it showed, I don’t know, how life is here ‘cuz it’s more peaceful and quiet. (Sarah)

**Figure 7.**
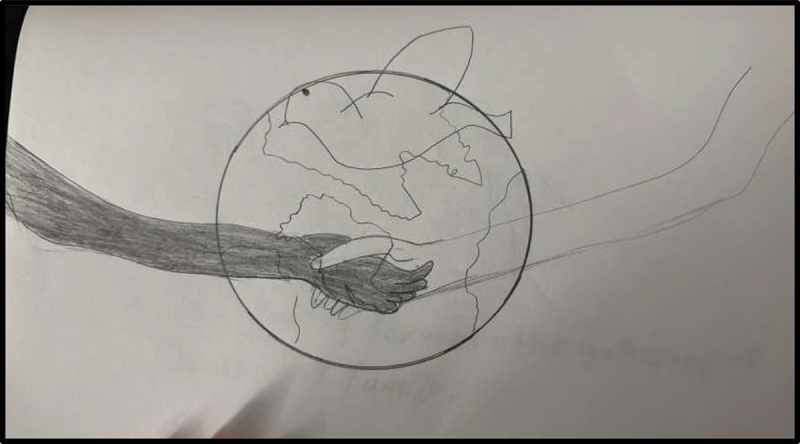
Cece, submitted in response to the prompt “how would you describe your life in the US to a friend back in your country of birth?”.

**Figure 8.**
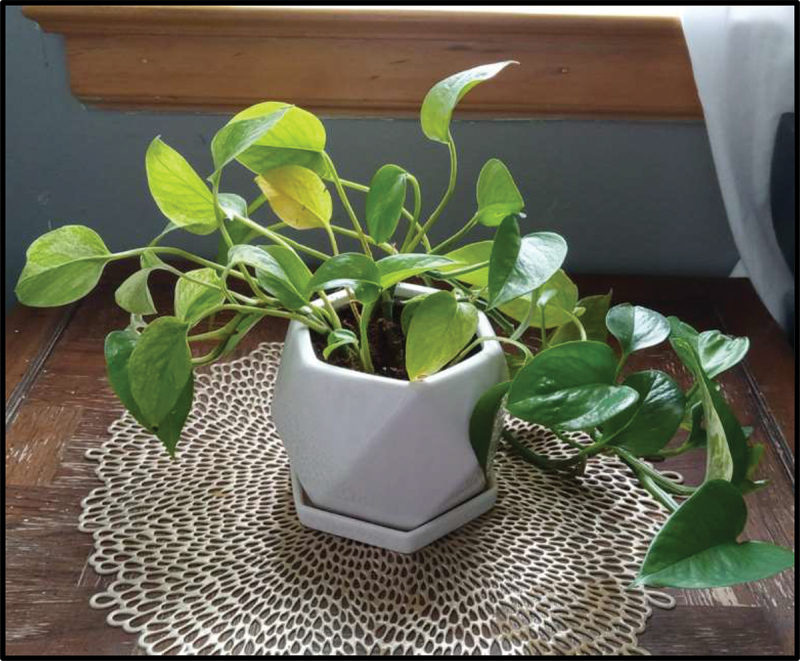
Jasmine, captioned “plants bring me relaxation,” submitted in response to the prompt “what does wellbeing mean to you?”.

An emphasis on peace seemed to be shared by participants from both Iraq and Syria. To represent her perception of life in the U.S., Cece shared a picture of a globe with a bird in front and two hands of different skin colours embracing, sharing that it symbolized “peace and safety” (Figure 7). Jasmine responded to the well-being prompt with a photo of a plant, saying it reminded her of “peace and joy” (Figure 8). For Julica, a sense of peace was most felt while listening to relaxing music and “looking at the roads;” she shared, “I get anxious in crowded places, you know. That’s why sometimes I like to go to calm places.” As these examples demonstrate, participants associated accessing quiet, natural public spaces with psychosocial well-being. Additionally, some emphasized how access to these spaces was a benefit of being in the U.S. as opposed to their home country: “everyone’s just like you and stuff but over here, everything’s just new. Like we don’t have stuff that we have over here. […] It’s just different, but I’d rather be here” (Sarah).

As reflected by participants, belonging within public spaces, including parks, museums, zoos, libraries, and schools, was not just mediated by their access to these spaces but by their ability to escape feeling overly observed or judged within them. For example, Sarah showed a picture of a water park (Figure 4) and described it as the space where she felt the greatest sense of belonging:

Nobody’s worried about what you’re doing. You’re just living your best life. And that’s what I like. Because you can be anywhere [and] people can be judging. I hate that. But, when you’re at a water park, nobody’s looking at you, they’re just living their best life and stuff!

Being in an outdoor space where the public coexisted in a way that minimized judgement and created a communal sense of joy defined Sarah’s idea of “living one’s best life,” a phrase that was repeated by several participants throughout the focus group discussions.

**Figure 9.**
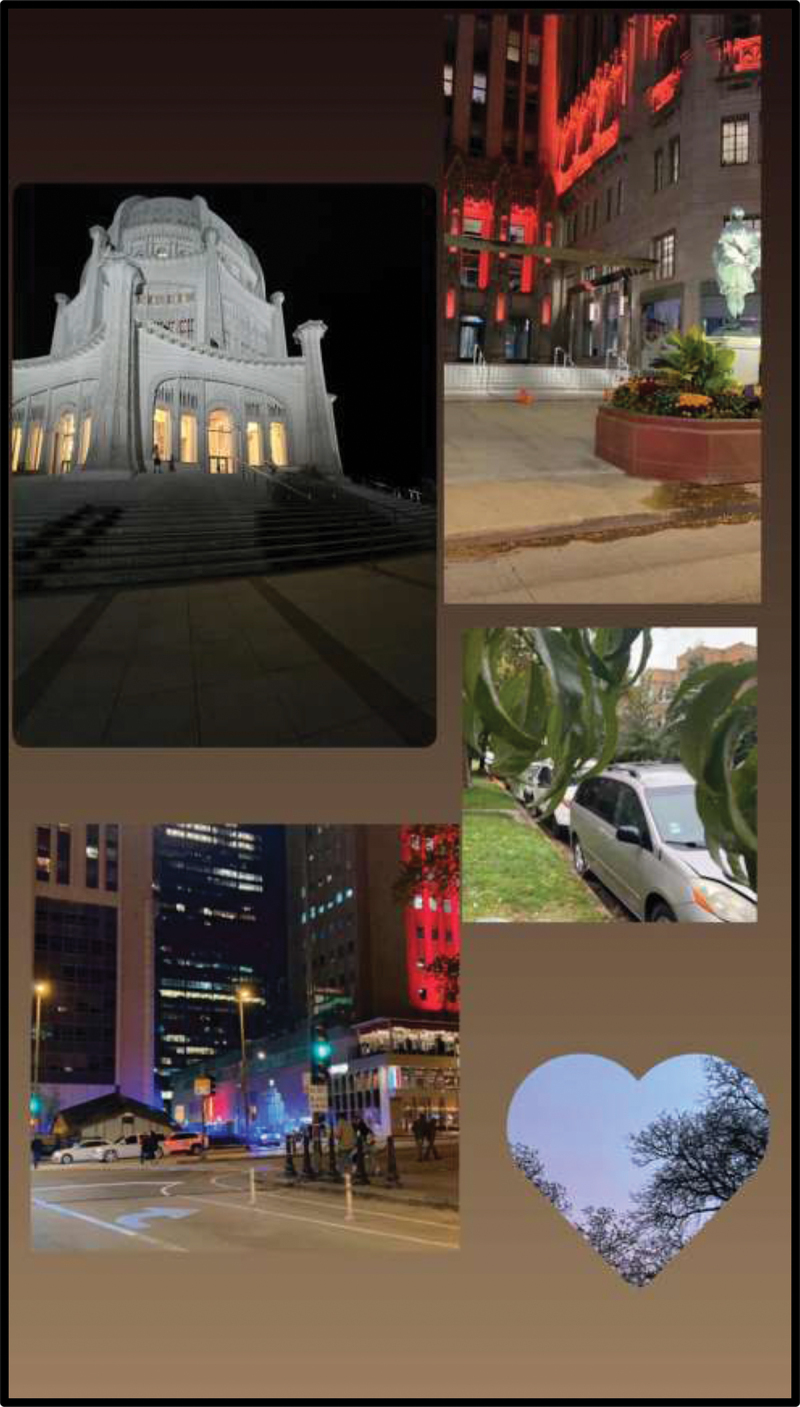
Soso, submitted in response to the prompt “how would you describe your life in the US to a friend back in your country of birth?”.

In a separate small group discussion, Julica described the precarity of retaining one’s “best life” in America from a socioeconomic standpoint, explaining how “some stores in America here are really expensive and you know, if all your money is gone, you can definitely go homeless and have no home to stay in.” In addition to inclusive treatment by those sharing public spaces, participants valued feeling a sense of security within their broader public environments, with housing and city infrastructure as recurring themes in their photos and discussions. Victoria shared her appreciation for the prevalence of traffic signs in the U.S., emphasizing that walking in the streets made her feel “safety.” In response to how she would describe her life in the U.S. to a friend back in her country of birth, Soso shared her love for Chicago’s “skyscrapers” and “big buildings,” and highlighted the “beautiful” public interfaith temple wherein she felt “warm and safety” (Figure 9). One participant, Julica, shared a photograph of her backyard with the caption “Why I belong here” (Figure 3) in response to the prompt about what makes her feel as though she most belongs in the U.S. She explained, “I love the buildings you know, and there is also like a backyard where […] you can like, plant stuff.” She compared the buildings in Chicago to those in her home country, proclaiming that “there are better buildings here in America … and you get to have an apartment all by yourself, you know, or like a mansion, or a house.”

### Emphasis on intersectional inclusion in public spaces

**Figure 10.**
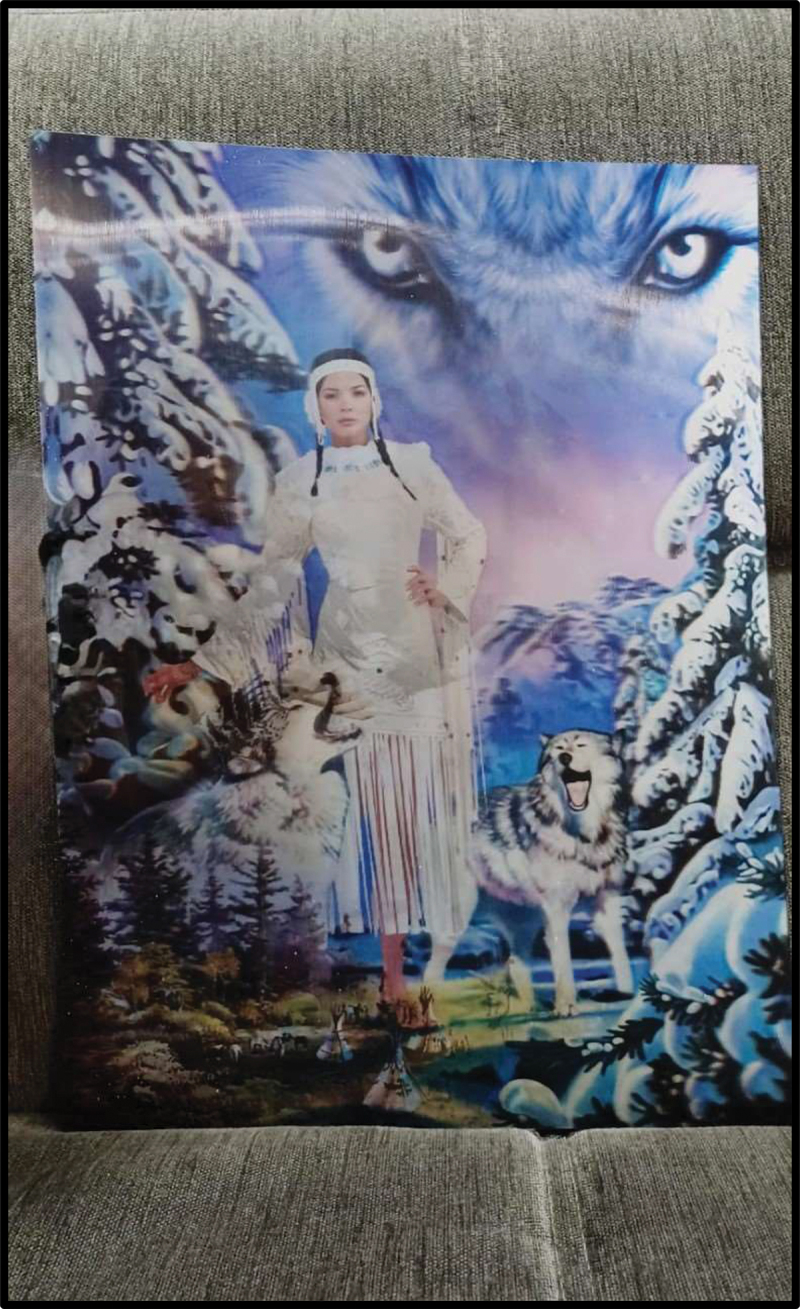
Julica, captioned “native American girl 3D hologram frame,” submitted in response to the prompt “what do you want people to know about you?”.

**Figure 11.**
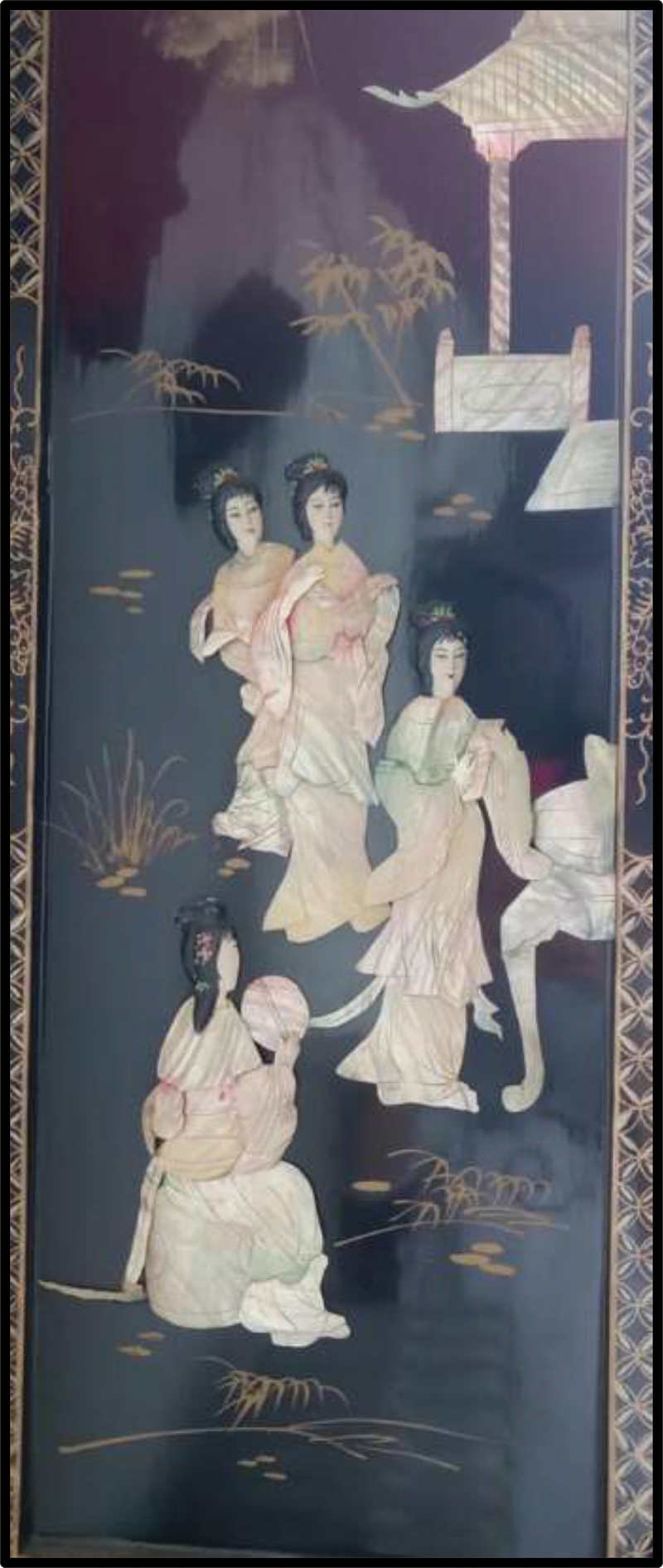
Jasmine, captioned “what I like about Chinese people,” submitted in response to the prompt “what do you want people to know about you?”.

Participants further utilized public spaces as a vehicle to explore well-being and belonging by emphasizing the intersectional systems of marginalization that shaped their lives and those of others in their families and communities. While presenting their photos, they compared their experiences in public spaces across contexts, prompting them to reflect on both cultural dissonances and strengths arising from migration and acculturation. In their subsequent data analysis and poster designs, they honed in on topics of justice and accountability and advocated for inclusion in public spaces in terms of identity markers including age, disability, gender, sexual orientation, and religion. Participants expressed appreciation for different cultures in their photos (Figures 10 and 11) and demonstrated a fervour for dismantling bullying in all forms and a longing for justice and accountability in situations of exclusion and injustice.

While positive experiences in public spaces contributed deeply to participants’ sense of well-being and belonging, they also shared experiences of Islamophobic discrimination in their broader Chicago communities. Sarah described one experience while also acknowledging the omnipresence of such harassment:

It can happen anywhere. I’m a hijabi so like, people are not always used to that. And like, they can be saying anything. And like, this one time, I was just walking and someone called me and they were like “bomber.” They’re just weird. But I don’t let it get to me and stuff.

Although Sarah exhibited resilience and was able to brush off the negative comments, her recollection of the discrimination she faced as “a hijabi” highlights a major barrier to participants’ sense of belonging in public spaces at the intersection of religion, gender, and race/ethnicity. As a physical marker of these intersecting dimensions–and a deeply important symbol of identity and culture for some Muslim women and girls–Sarah’s hijab may have also drawn attention to her religious background, creating a dissonance between her connection to her cultural community and her increased vulnerability to harassment. Julica, who also wore a hijab, highlighted similar themes: “sometimes people in America, you know, treat you bad for your religion […] they should respect each other, they should treat other how they want to be treated, you know, not being racist, or not being offensive towards each other.”

**Figure 12.**
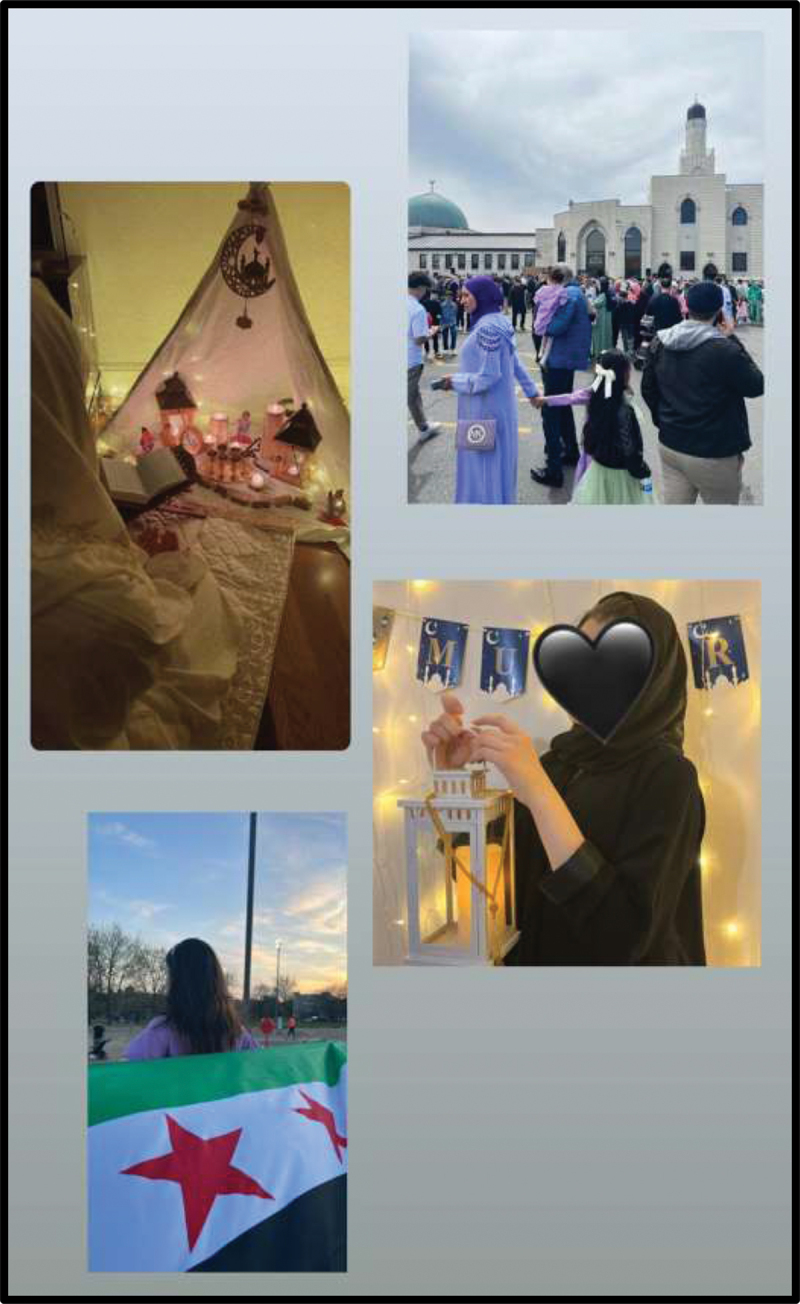
Soso, submitted in response to the prompt “what does belonging mean to you?”.

Cece and Soso, who had arrived in the U.S. more recently than the other participants, had a different perception of the level of racial discrimination present in their communities. Soso shared that when she was leaving Syria and Jordan to come to the U.S., she “thought that in America, ‘oh my gosh … I am coming to a city or a place that is full of racism/ او ماي غاش، انا جاي على بلدة أو مكان كله عنصرية.” For her, the Muslim community was a source of comfort. While she expected to “miss my country… miss my religion,” she felt belonging by “going to masjid [the mosque]” and celebrating Ramadan. To represent the importance of her Islamic religious practices, traditions, and places of worship, Soso depicted them in a collage of photographs responding to the prompt on belonging (Figure 12). Cece echoed these sentiments and advocated for intersectional inclusion beyond religion: “I accept everyone, you are gay, you are attracted to other people, it’s okay, as long as everyone is by themselves, no bullying, because everyone has their own culture, traditions, and religion/اوك ممكن صح أنا مو ضد بس انا بس ، هما الأشخاص حرين بنفسهم لما … بتقبل الكل، انت مثلي، انت عندك ميول لناس ثانية عادي، طول كل واحد بحاله، ما فيه تنمر، لأنه كل واحد اله ثقافته عاداته دينه.” Across all sessions and among all participants, the advocacy for justice and accountability spanned various dimensions as participants called for systemic changes to ensure equal opportunities and protection for marginalized groups.

Participants also perceived discrimination within their families at the intersection of age and gender. They described restrictive household rules placed on them by family members that often made it more challenging to access the spaces they needed to feel affirmed and live their “best lives.” For example, Jasmine shared:

I have one little brother, and it’s kinda annoying sometimes because they say, well I heard, like, a girl is supposed to be the one at home by herself, cooking and cleaning all the time, and the boys are the ones that get to go outside and live their best lives. And it’s kinda not fair. Because who are they to tell us what to do just because we’re women or just because we’re girls? Like that doesn’t mean that we can’t live our best lives. Like, either way, I won’t listen to them because if people try to tell me “oh you’re a girl, you have to do this or that because, just because you’re a girl,” I won’t listen to them because who are they to tell me what to do? I’m going to live my best life.

Sarah perceived similar gender-based injustice within her home, sharing that her caregivers “expect me to clean, but my brother doesn’t do anything, he’s just like, at home chillin’ or playing on his phone or PlayStation, but he never helps out. It’s always my job. It’s sexist.”

The struggle against restrictive household rules unveils the delicate balance participants had to navigate between personal aspirations and cultural expectations, including the influence of migration. Participants acknowledged that parental restriction of their access to public spaces may be coming from a place of protection, especially if they were aware of adolescent girls’ exposure to Islamophobic harassment and discrimination in their new context. As Jasmine illustrated: “There’s so many protective parents, but sometimes they’re like, really overprotective. Like you just wanna hang out with your friends or something.” Meanwhile, Sarah emphasized how her peers often had different expectations in terms of their movement in public spaces: “my parents are Muslim, so they’re like, strict about this stuff. And all my friends are like, free.” In describing her friends as “free,” Sarah seemed to imply that she did not see herself that same way, resulting from the strict rules that were enforced by her parents and perceived to be guided by their religious beliefs or cultural norms.

In addition to participants’ emphasis on perceived sexism within their families–and the impact this had on their own access to public spaces–participants advocated for greater inclusion of individuals with disabilities within their Arab communities. For example, Soso explained that back in Jordan and Syria, children with disabilities were isolated from other students in an expensive private school, while in the U.S. it was “okay to get into the high school of his brother and sister.” Both Cece and Soso felt that excluding peers with disabilities was the norm in Arab countries, which led to bullying and stigmatization. Cece explained the way she imagined people with disabilities should be treated, emphasizing the role of the family:

I meant that, the family should take responsibility, when the family takes care, and they don’t distinguish between children, for example a child with special needs and another child. The family doesn’t allow people outside to bully their child … they treat their child, it’s not empathy, but treat their child like a normal person, not special treatment. But we in the Arab countries make them feel that they are providing them with special treatment.

انه قصدي، انه، المفروض بتتحمل المسؤولية العيلة، لما يكون الاعتناء من العيلة وما يكونوا فارقين بين الطفل مثلاً ذوي الاحتياجات الخاصة والطفل الآخر. ما يسمحوا للناس البرا انه يتعرض للتنمر. حتي لو يتعاملوا معه مو انه كتعاطف، لا كإنسان طبيعي. مو معاملة خاصة. بس احنا عندنا في البلاد العربية انه لا بيحسسوا انه معاملة خاصة

Soso echoed the sentiment that family support was crucial in fostering belonging among youth with disabilities, and she also acknowledged her own role in supporting peers: “I hate when someone is bullying someone else, especially a person with special needs. If I saw someone with special needs, I might leave what I am doing and go to them and play with them and have fun with them/اذكرت شيء كنت بدو قوله، اللي هوا بكره مثلاً حدا يتنمر على الثاني وخصوصاً ذوي احتياجات خاصة، اذا لو شفت حدا عنده احتياجات خاصة انا ممكن اترك القعدة اللي انا قعدا عليها، أو الموضوع اللي اقاعدة فيه واروح عنده عادي والعب معه، اقعد العب معه واتسلي معه.” Participants’ shared emphasis on inclusion of peers with disabilities demonstrates their passion for justice and attention to accountability within their community, signalling a desire for efforts to foster belonging beyond their own.

**Figure 13.**
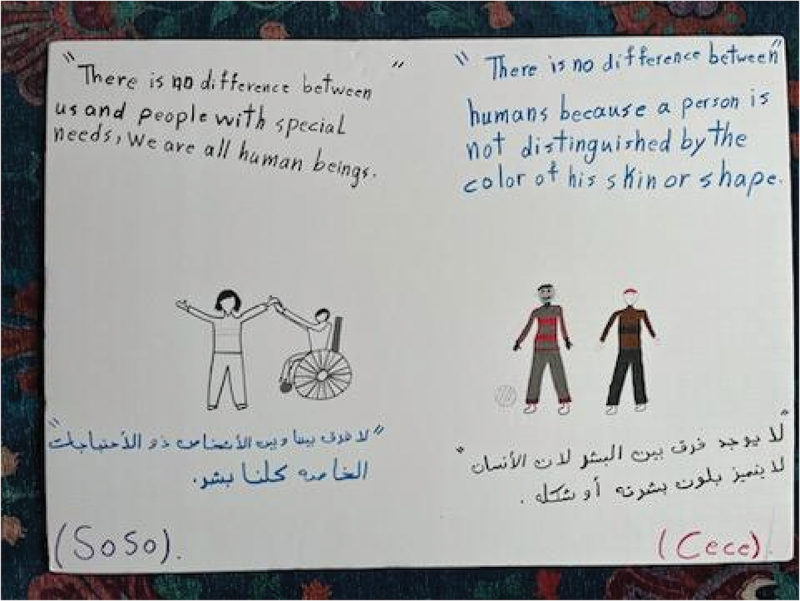
Cece and Soso, action plan poster.

**Figure 14.**
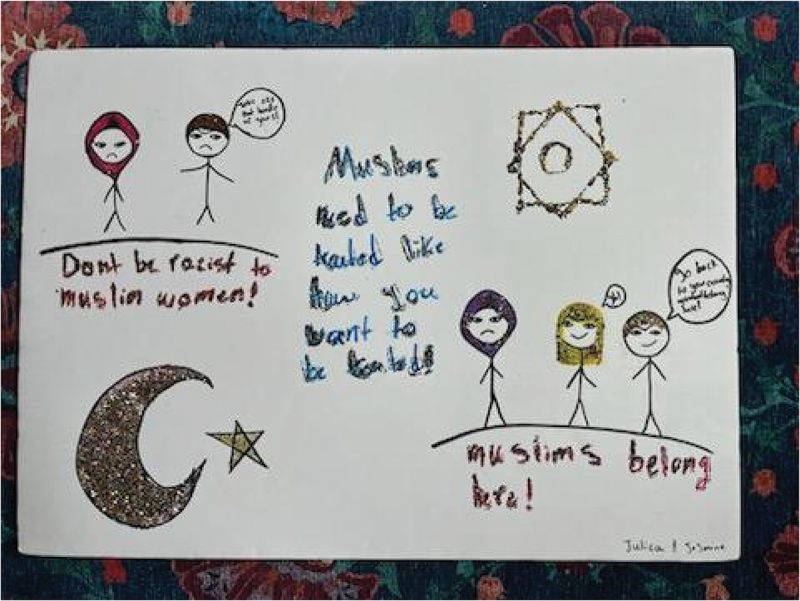
Jasmine and Julica, action plan poster.

**Figure 15.**
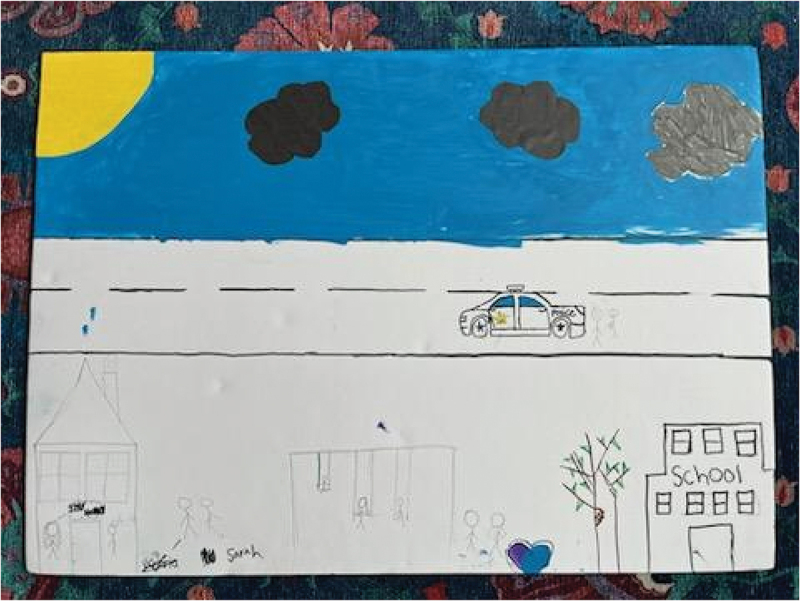
Sarah and Victoria, action plan poster.

Participants reflected their advocacy for intersectional inclusion in the posters they created for their PhotoVoice “action plan.” Cece and Soso’s poster showed images of people with different skin colours, including a wheelchair user, along with text in both English and Arabic reading “there is no difference between us and people with special needs, we are all human beings/لا يوجد فرق بيننا وبين ذوي الاحتياجات الخاصة، فكلنا بشر” and “there is no difference between humans because a person is not distinguished by the color of his skin or shape/فلا فرق بين البشر لأن الإنسان لا يتميز بلون بشرته أو شكله” (Figure 13). Meanwhile, Julica and Jasmine’s poster depicted images of people harassing hijabi women with xenophobic remarks such as “go back to your country!”; they countered these drawings by centring statements such as, “don’t be racist to Muslim women,” “Muslims need to be treated like how you want to be treated,” and “Muslims belong here” (Figure 14). Finally, Sarah and Victoria’s poster illustrated a neighbourhood with people enjoying swings on the park, a home, a school, and a police car on the road (Figure 15), representing a sense of belonging and safety in their community.

### Role of schools in facilitating belonging and access to public spaces

Participants conceptualized their schools as microcosms of public spaces wherein they valued friendships with students from diverse backgrounds and supportive teacher relationships, yet also experienced discrimination in the form of bullying. They also emphasized the resources provided by schools that they perceived to expand their access to public spaces and to facilitate their well-being.

**Figure 16.**
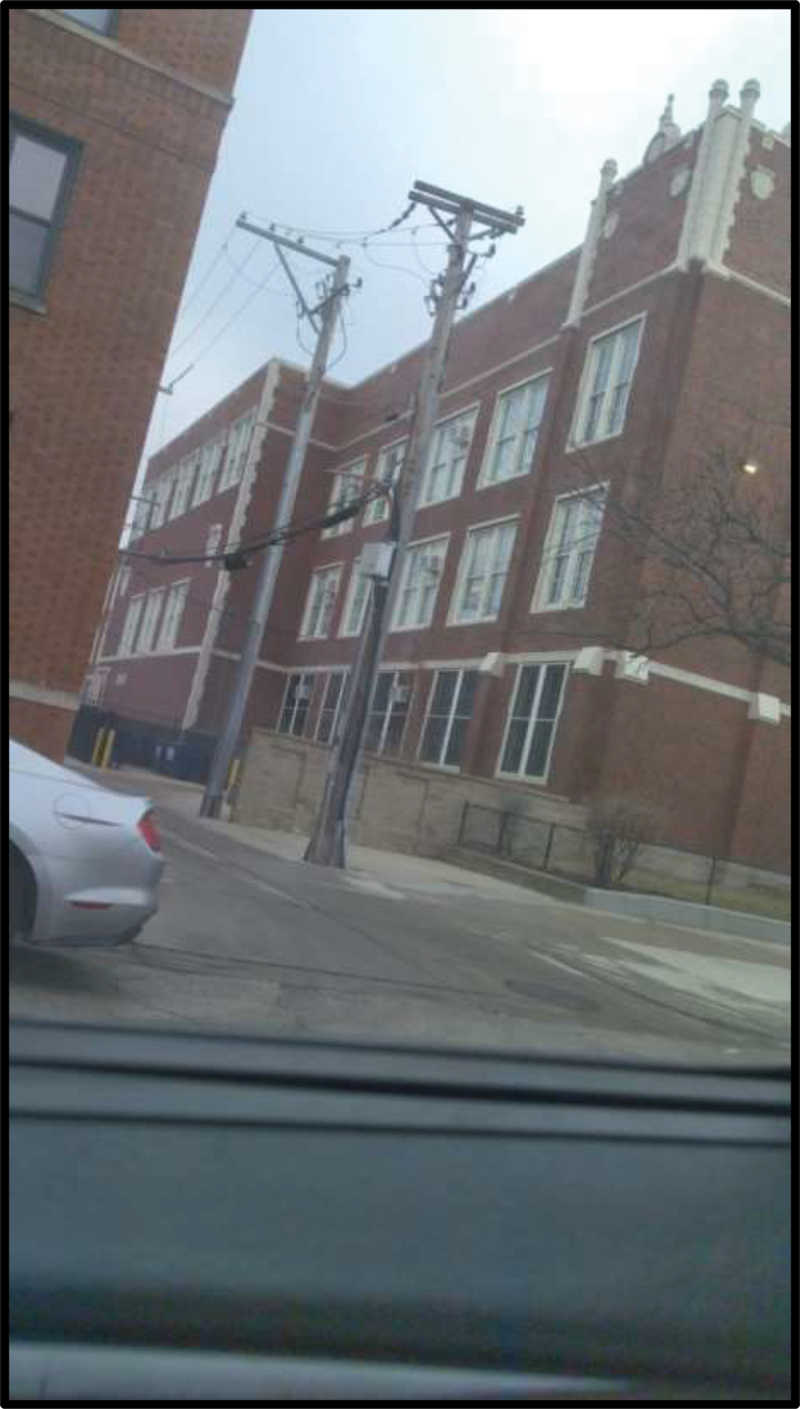
Jasmine, captioned “the buildings are very tall and wide,” submitted in response to the prompt “how would you describe your life in the US to a friend back in your country of birth?”.

Several participants expressed appreciation of their schools’ diverse student body composition, which enabled cross-gender and cross-cultural peer relationships and marked a difference from their school experiences in their countries of origin. For example, when asked how she would describe life in the U.S. to a friend in her home country, Jasmine shared a photo of her school (Figure 16) and explained:

I felt like it was very important for me to put it there because our school is very different–like different people from different countries, different religions, different languages all together even though we’re all very different. [… What makes it good is] the fact that you get to hang out with different people from different places. Like you get to experience … Like even if you didn’t go to where they’re from, you still get to hear about it.

At the same time, multiple participants described being the targets of bullying by peers in their schools; Sarah described the bullies in her school as “childish” and wanting “to get as much attention from everyone by being rude to other people.” Meanwhile, Jasmine emphasized the impact that bullying had on her self-esteem, explaining that the bullies in her schools were boys who “would look at you and like, make those comments. And they’ll be like, laughing and giggling to the side. And then just like, makes you feel so bad about yourself.” Participants sought support from trusted adults when treated poorly by peers. For example, Julica reported her experience of being bullied to her mom and Sarah to her teachers; both shared that their school took action to put a stop to the bullying.

However, not all teachers were perceived to be helpful in addressing the unwanted behaviour. Jasmine discussed the behavioural management practices of teachers, recounting, “Some of the teachers just yell at them, then they stop for like five seconds, and then again, they start again being so immature.” At times, these experiences inadvertently became distractors from participants’ learning in the classroom—or even restricted their opportunities to engage in public spaces via field trips. Sarah shared an instance of collective punishment for the behaviour of bullies: “We were gonna go to Six Flags this year, but because of the boys and what they did, they canceled it ‘cuz they’re immature, so they wouldn’t trust them going to Six Flags.”

Despite social and economic barriers, schools were largely considered affirming environments that facilitated access to broader, enriching experiences in their surrounding neighbourhoods. For example, one participant fondly shared about a field trip wherein her school took her class to visit animals (“cows … and wolves!”) after the related curriculum in the classroom: “Sometimes if you learn about something, about a place, the school takes you there! That’s what happened to me in fifth grade” (Victoria). When asked by the co-facilitator if that experience made her feel a sense of belonging, Victoria affirmed, indicating the value of school efforts to broaden newcomer students’ interactions with public spaces. At the same time, these field trips sometimes required students to cover part of the cost, potentially making them inaccessible to some. Jasmine described her school’s “learning celebration” wherein students were split into teams and earned points for different classroom activities. While the winning team was awarded a trip to the “cinema by our school,” students still had “to pay 10 dollars for it.”

Another resource provided by the school that participants deemed significant to their well-being were bus cards. Victoria explained, “They give you [a] bus card and in Iraq, they don’t give you [a] bus card. They just let you walk home.” When asked what she thought about using the bus system in Chicago for transportation to and from school, Julica shared, “I think this is great because in Syria, you definitely don’t have that.” As compared to their home countries, participants underscored the way schools created opportunities for them to interact with public spaces in Chicago, whether directly through field trips or indirectly through increasing their freedom to move around the city. Explaining how important the bus cards were, Victoria said, “If it’s like cold weather, and if it’s hot, so they give you a bus card, and just like, I am grateful for school.”

In addition to providing participants with resources and direct opportunities for interacting with public spaces in Chicago, schools played a critical role in promoting participants’ general wellness, contributing to their confidence and sense of belonging in the U.S. One important school resource that was mentioned multiple times in focus group discussions was schools’ contributions to food security for participants. Julica explained, “So we talked about school lunch where they provide you with free food and cook for you. But in Syria, they don’t do that … So in America, they help you.” Another participant, Victoria, explained the significance of a school-based programme which provided fruit and vegetables for families, saying, “They put it in front of school, if they leave, if you want to, you can grab them.” The anonymous nature of this system was important to Victoria, as it allowed schools to support students and their families without making them feel ostracized or ashamed of needing help. Victoria also went on to compare schools’ role in food security for students in her home country, saying “In Iraq, they don’t do that … schools, they can’t pay that much to give vegetables to children.” By comparing the resources available within their Chicago school system and their home countries, participants seemed to emphasize the vital role of U.S. schools in ensuring their material needs. While none of the participants explicitly stated their own use of this food security programme, they expressed feeling supported and comforted in knowing that they had access to these resources.

Supportive relationships between students and school staff were also considered affirming for participants. Sarah emphasized such a dynamic with some of her teachers: “I just love them. And like, if I want to tell them about something, they’re gonna keep to themselves. You can trust them, and they help a lot.” When asked what kind of support the school provided to foster her belonging, Julica responded, “Yeah, so first, when I needed help, of course, the teachers helped me.” She continued, “I am also grateful for the janitors [… and] for the lunch ladies and lunch men.” Jasmine expressed disappointment about how her favourite teacher was “doing such a good job” at offering her support that the district had pulled the teacher “to go to another school and help other teachers with their jobs.” Acknowledging the frustration within school spaces while also detailing the important relationships that are formed in this setting not only highlights existing challenges but also emphasizes the potential impact of a supportive and understanding educational environment.

Overall, schools played an important role in mediating participants’ opportunities to engage in public spaces. Participants referred to schools as a space of coexistence of diverse groups and a centre of friendship. They emphasized a need to ensure that school spaces remain affirming, free from judgement, bullying, and collective punishment.

### Language as a critical barrier and facilitator

Throughout the sessions, participants were encouraged to speak in both Arabic and English. Two participants, Cece and Soso, spoke mostly in Arabic as that was the language in which they felt the most comfortable expressing themselves. As the most recent newcomers among the group, with both resettling in the United States from Syria in 2022, they were less fluent in English than the others. At times, Cece and Soso would pause their English speaking and switch to Arabic to ensure their perspectives came through clearly. In this transition, their contributions to discussions were much more robust and emotionally driven in comparison to their contributions in English. During focus group discussions, the role of language and language learning became clear as a critical barrier to and facilitator of relationship building and belonging within the school and community context. Cece and Soso also used both Arabic and English text on their action plan poster, which centred on the inclusion of people with intersectional identity dimensions (Figure 13).

Participants emphasized the importance of developing English language skills for promoting their sense of belonging and their ability to navigate Chicago and its public spaces. For some participants, school and the relationships they built there were the most critical factors for improving their English. Julica shared why learning English was so important to her sense of belonging, explaining,

I wanted to learn English so quickly and so bad because you know, other people around me spoke it and I was like, I was really confused about what they were talking about, you know? Yeah, it was really difficult for me, because you know, it’s a second language, and learning a second language can be hard, especially when you’re an English learner.

The ability to feel connected to the people around her through a shared language was deemed crucial for developing a sense of belonging, particularly within Julica’s school community. Julica also shared that school allowed her to meet new people who she could practice her English with, elaborating, “I could also learn English from my friends.”

Moreover, school was a place where participants described having to navigate the challenges of acculturation as it was linked to language. When further prompted if she ever felt “in between” her country of origin and Chicago, Jasmine shared that she felt this way most in the school context. She explained, “When I’m talking with my friends, I feel like I’m Muslim … But like, at home like I call my friends and family, I feel like the opposite, because like, they’re in Syria and I’m in America now, because like, it’s like, they’re in Syria and I’m here, because I talk English now.” The use of English and Arabic, whether with American friends or Syrian family, influenced this participant’s perception of her identity as she navigated feelings of belonging between her various cultural communities.

Participants also spoke of the difficulty in retaining both English and Arabic. The loss of their native language, at times, made them feel disconnected from the local community of their country of origin. As Jasmine expressed:

Other kids from Syria or from any Arab country, they come here to America, when I hear them speak, um, Arabic, sometimes I feel like I don’t belong with them anymore. Because I feel like I belong in America more than Syria now because of the language, you know? Because I speak more English now than Arabic. So yeah, I’m kinda forgetting my Arabic. It’s disappearing on me.

Participants’ shared experiences around the role of language indicate its important connection to belonging and self-expression in different spaces, including among peers in school settings. Participants expressed that as their English language learning improved, they felt more capable in English-speaking spaces. Unfortunately, however, this seemed to come at the expense of heritage language preservation, resulting in diminished belonging among Arabic-speaking newcomer students and those who had been in the U.S. for longer.

As co-researchers, participants led the data generation and analysis process by presenting photos that represented their ideas of well-being and belonging, notably including numerous public spaces and their schools. Throughout the process, they promoted inclusion and emphasized both their Arabic and English language abilities as key resources that connected them across cultures and strengthened their feelings of belonging among family and peers.

## Discussion

Adolescence is a time of significant developmental change that can impact mental health and well-being, and for adolescent refugees resettled in the US, these changes can be compounded by acculturation challenges in their new homes. Utilizing the PhotoVoice methodology, SALaMA EMPOWER serves as a conduit for adolescents to actively engage as co-researchers, lending their voices, insights, and priorities (Budig et al., 2018; SALaMA, 2021). Findings from this study centre the lived experiences and community priorities of six adolescent girls who arrived as newcomers to Chicago, Illinois from Iraq and Syria during the last 10 years. The central thread throughout the photos and experiences participants shared was the importance of affirming public spaces, notably including schools, in facilitating their well-being and belonging. The findings presented here were gleaned from researcher-conducted data analysis—including and building upon the themes participants themselves emphasized when coding and analysing findings from their own focus group discussion data. Further, the importance of affirming public spaces as a vehicle for belonging and thus a community priority was reflected in participants’ codes (see Table I), diagrams, photos, and action plan poster designs (see Figures 1–15), alongside their perceived barriers to inclusion. Notably, the findings from this study reflect the agency and power of refugee youth; protected by pseudonyms, the participants leveraged the programme’s focus on belonging and well-being to bring attention to intersectional forms of discrimination within their families, schools, communities, and public; to call on their community members to be more accepting and respectful of identity-based differences; and to highlight the critical role of language in their acculturation process. Findings also have implications for schools, with analysis revealing the school setting as an institutional space causing frustration, but also a space wherein programming and resource distribution—i.e., field trips, language support, bus cards, and food—can facilitate greater inclusion in both schools and public spaces for newcomer youth.

Findings from this paper extend the growing literature base around the importance of refugee youth’s access to safe and affirming public spaces in urban resettlement contexts (Chen & Knöll, 2022; Karamese, 2023; Macaulay, 2023; van Liempt & Kox, 2023; Ziersch et al., 2023). In addition, results add to the vital body of evidence emerging from the longitudinal SALaMA project by centring youth voices in defining the collective change they want to see in their communities. The SALaMA EMPOWER programme described in this paper adds the important perspectives of resettled youth living in Chicago-one of the largest urban contexts in the U.S. Using PhotoVoice as a methodology, EMPOWER bolsters our understanding of the requisite supports and services that can facilitate a smoother transition and successful integration of students from conflict-affected countries in the MENA region into the United States and their new educational environments. Previously, SALaMA co-researcher youth who participated in the same PhotoVoice programme in the Detroit Metropolitan Area highlighted the strength of their region’s Arab enclave in facilitating healthy resettlement and acculturation (Smith-Appleson et al., 2023); in comparison, participants in Chicago felt belonging among their cultural communities but particularly valued the diversity and inclusion of people with intersectional identity differences in their community and public schools. A qualitative study conducted in Istanbul with migrant youth from Syria described such spaces as “comfort zones,” or spaces wherein migrant youth “feel less labeled, and they have encounters with many different groups of people, and as such, they feel more integrated into society and have more of a sense of belonging” (Karamese, 2023, p. 144). “Comfort zones” resonate with the way participants in this paper perceived certain community spaces including water parks and beaches, and the value they attributed to a sense of calm or peace within these spaces that contributed to their well-being. Meanwhile, Karamese (2023) defined “judicial spaces” as those in which migrant youth recognize uneven power relations, or those which feel like “symbolic courts in [which] people are being judged and tried” (Karamese, 2023, p. 145). Judicial spaces were reflected in participants’ experiences of bullying and discrimination in their schools and communities. Findings from this paper contribute to Karamese’s (2023) spatial framework for belonging. Further, they underscore the importance of engaging community actors to bolster school and public spaces as “comfort zones” that promote belonging and inclusion in their policies and practices.

Against the backdrop of a socio-political landscape marked by escalating Islamophobia and anti-Arab sentiments in the United States, the imperative of ensuring inclusive access to public spaces takes on a heightened urgency (Rehman & Hanley, 2023). The lived experiences of newcomer students originating from Arab countries are situated within global systems of power and oppression, shaping their intersecting identity dimensions, perceptions, and challenges in profound ways. For the participants of EMPOWER, contributing their lived experiences as research also extended to advocating for the inclusion of others who held marginalized dimensions of identity. The study’s findings underscore this communal ethos; Participants, cognizant of their own struggles, also recognized the broader societal imperative to embrace and empower others. Moreover, the study illuminates the potential peril of unchecked harassment and discrimination, which can quickly escalate into violence, reinforcing the urgent need to cultivate a culture of belonging and acceptance within society at large (Grinshteyn et al., 2022). These insights highlight the intricate tapestry of challenges and opportunities that confront newcomer students from Arab countries, urging a comprehensive and nuanced approach to fostering inclusivity and belonging (Gillespie et al., 2022; Smith-Appleson et al., 2023; Stark et al., 2020).

The growing use of PhotoVoice methodology to explore these themes among newcomer youth is promising, as the use of photography centres participants’ lived experiences and offers a way to engage regardless of shared language ability (Miled, 2020; Springer et al., 2023). Via photos, participants were simultaneously able to navigate their emerging intersectional identities and realities in the U.S. and honour memories of their home countries and cultures (Feen-Calligan, Grasser, Smigels, et al., 2023). Further, the community- and school-based galleries showcasing participants’ PhotoVoice materials encouraged greater recognition of resettled adolescent girls’ contributions to their local Chicago community, promoted their agency and artistic expression, and facilitated valuable insights into approaches practitioners can take to implement relevant programming and policies that support refugee well-being and inclusion.

At the heart of efforts to cultivate belonging for newcomer students lies the pivotal role of educational institutions, particularly schools (Kalchos et al., 2022; Killian et al., n.d.). Serving as reflections of broader societal dynamics, schools play a critical role in shaping students’ integration experiences. Instances such as the cancellation of field trips due to the actions of a few students offer a poignant glimpse into systemic issues within educational settings, where collective punishment often undermines principles of fairness and inclusivity. To effectively address these challenges, schools must embark on a multifaceted approach, prioritizing inclusivity from the outset. This entails creating environments where all students feel embraced, valued, and empowered to thrive. A cornerstone of this effort lies in supporting language learning initiatives, recognizing language not only as a means of communication but also as a powerful vehicle for identity expression and cultural connection (Gillespie et al., 2022; LANGUAGE Integration Barriers, n.d.). Findings from this study underscore the significance of providing opportunities for learning in public spaces, particularly in urban settings where access may be limited (Larson et al., 2016). Additionally, involving parents and families is paramount, as perceived safety concerns and cultural norms may influence students’ engagement in public spaces (Kelly & Wakabayashi, 2020; Liu & White, 2017). As co-researchers, participants in the study identified educators and mentors as pivotal agents in shaping their acculturation experiences; newcomers explicitly noted how school-based staff fostered belonging, destigmatized food and transportation supports, and developed student-teacher relationships built on trust. Participants’ affirmation of the critical role of school staff in providing holistic support and guidance, beyond academic instruction, adds to a growing literature base that emphasizes such contributions to short- and long-term student well-being outcomes (Allen et al., 2021; McDiarmid et al., 2022; Uslu & Gizir, 2017). By recognizing and addressing the multifaceted needs of newcomer students, schools can serve as catalysts for positive social change, fostering environments where all students feel a profound sense of belonging and agency (Bennouna et al., 2021; McDiarmid et al., 2023).

Our results provide evidence for multiple junctures at which schools can provide greater support for their newcomer students. First, our participants’ concerns that their families are restricting access to affirming public spaces due to gendered cultural norms around protecting girls establishes the opportunity for schools to engage in dialogue with newcomer families about city safety, particularly for young women. Providing parents and caregivers with information about how to navigate new urban contexts more securely may ease anxiety and introduce conversations surrounding the particular protection of adolescent girls, without overstepping boundaries. Moreover, the theme of intersectional inclusion and our participants’ repeated critiques of some teachers’ unjust discipline practices leads to our recommendation that schools strengthen teacher competence and training around restorative justice practices for the classroom. Some students placed their trust in teachers, indicating that strengthened teacher-student relationships are imperative to fostering an affirming learning environment.

Our evidence supports the need for school-based interventions that are particularly concerned with peer-to-peer level interaction. Specifically, programming that facilitates peer tutoring between native English speakers and newcomer students may be beneficial for English language learning for newcomer adolescents. Our participants noted that one of their key methods for language learning involved social dialogue; their capacity to form friendships across diverse groups of students at school facilitated English language skills. In a review of studies that evaluated peer tutoring for English Language Learning, Bowman-Perrott et al. (2016) found that, across English proficiency levels, peer tutoring consistently leads to gains in academic language skills and provides social benefits for learners. Participants’ stated experiences in our study resonate with this finding, as well as with other prior research highlighting how newcomer adolescents’ agency in their new community is deeply tied to social relationships, and primarily peer friendship (Verdasco, 2019). Our results emphasize the need for schools to employ more peer-based learning models to both support newcomer students’ academic language learning and the growth of social connections across their student body.

Finally, schools should maximize their ability to provide opportunities for students to explore the public spaces surrounding their school communities. Given the thematic emphasis on public spaces and language as dual facilitators of well-being and belonging, schools might consider expanding opportunities for language learning outside of the classroom in alignment with a growing evidence base on the value of immersive and community-based language learning for immigrant students (Chan, 2023; Clifford & Reisinger, 2019; Kukulska-Hulme et al., 2017). Educational and motivational field trips, subsidized outings, and educational resources about local events or spaces all have the potential to support newcomer students who rely on affirming public spaces as a way of engaging with their new environments. In these spaces, educators can leverage the “linguistic landscape as a pedagogical resource,” encouraging multilingual students to analyse public signage, notices, and advertisements to develop literacy skills and symbolic competence in their communities (Back, 2016; Kukulska-Hulme et al., 2017; Sayer, 2010). Schools should continue to dedicate their resources towards providing students with food, bus cards, and language support to facilitate such learning and to promote their well-being and sense of belonging within both schools and Chicago urban spaces more broadly.

### Strengths and limitations

Our analysis and study design must be framed with consideration to their strengths and limitations. Primarily, our participatory study design and participants’ engagement with photography and qualitative research analysis was a significant strength of this study. Participants were empowered as co-researchers in our methodology, and our analysis centred the priorities and needs that emerged from their photographs, focus group discussions, and action posters. Participants were able to take ownership of the study by supporting the curation of their final PhotoVoice exhibit at a local community-based support organization and a gallery event at a CPS high school, witnessing the transformation of their time into tangible work that they could share with their community.

The sample demographics of our small, all-girl sample were both a strength and a limitation for this study. The intimate size of group discussions and existing relationships between participants encouraged lively conversation and allowed them to introduce vulnerable topics such as economic precarity, bullying, and discrimination. While our study does not capture the perspectives of boy or gender minority newcomers, the all-girl space may have allowed participants to open up more comfortably about their frustrations with intersectional, gendered social and family norms, building group solidarity and trust as they nodded, laughed, and exclaimed in agreement with each other.

The programme location, a public library, presented both a strength and limitation for our study. The library was a welcoming and familiar environment that was freely accessible to all participants, providing a neutral space for the participatory collaboration between facilitators and participants that our methodology encouraged. However, upon reflecting on the participants’ key focus on public spaces in our analysis, the physical setting of the sessions may have put public spaces at the forefront of participants’ minds as they conversed. Their emphasis on outdoor public spaces in particular may have been influenced by the timing of the programme; winter in Chicago is known to be harsh, and the sessions coincided with seasonal warming and the re-emergence of many people to public spaces.

Finally, our participants and study provide outcomes for the Chicago area and public school district context. While the EMPOWER programme was limited to students residing in this metropolitan area, many of our findings will be applicable in other urban contexts, particularly as school districts continue to receive more newcomer students from the MENA region.

## Conclusion

In conclusion, this study illuminates the multifaceted challenges and opportunities faced by resettled adolescent girls from the MENA region in Chicago. Engaging participants as co-researchers using PhotoVoice methodology revealed profound insights into their lived experiences and community priorities. While affirming public spaces emerged as central to their well-being and sense of belonging, the findings also highlight broader issues such as gendered cultural norms, unjust discipline practices in schools, and the importance of peer-to-peer interactions. These insights underscore the need for comprehensive, inclusive approaches in schools and communities to support the psychosocial well-being and integration of refugee youth. By centring refugee voices and employing an intersectional methodology, this study not only enriches our understanding of refugee experiences but also provides actionable recommendations for policymakers, educators, and practitioners working with resettled youth.

## Data Availability

The data that support the findings of this study are available from the corresponding author upon reasonable request.

## Note

1. In order to ensure participants’ confidentiality, participants chose their own pseudonyms before the beginning of the first session, which were then used instead of their real names throughout the program.
